# The antibacterial activity and therapeutic potential of the amphibian-derived peptide TB_KKG6K

**DOI:** 10.1128/msphere.01016-24

**Published:** 2025-05-19

**Authors:** Cristina Schöpf, Magdalena Knapp, Jakob Scheler, Débora C. Coraça-Huber, Alessandra Romanelli, Peter Ladurner, Anna C. Seybold, Ulrike Binder, Reinhard Würzner, Florentine Marx

**Affiliations:** 1 Biocenter, Institute of Molecular Biology, Medical University of Innsbruck, Innsbruck, Austria; 2 Department of Zoology, University of Innsbruck 27255, Innsbruck, Austria; 3 Institute of Hygiene and Medical Microbiology, Medical University of Innsbruck, Innsbruck, Austria; 4 Research Laboratory for Implant Associated Infections (BIOFILM LAB), Experimental Orthopaedics, University Hospital for Orthopaedics and Traumatology, Medical University of Innsbruck, Innsbruck, Austria; 5 Department of Pharmaceutical Sciences, University of Milan 9304, Milan, Italy; University of Nebraska Medical Center College of Medicine, Omaha, Nebraska, USA

**Keywords:** antimicrobial peptide, temporin B analog, *Staphylococcus aureus*, antibacterial activity, membrane activity, human epidermis equivalent

## Abstract

Antimicrobial peptides (AMPs) have great potential to be developed as topical treatments for microbial infections of the skin, including those caused by the gram-positive human pathogen *Staphylococcus aureus*. Among the AMPs, temporin B (TB) is of particular interest. This 13-amino-acid-long cationic peptide is secreted by the granular glands of the European frog *Rana temporaria* and represents a primary line of defense against invading pathogens. The objective of this study was to investigate the antibacterial efficacy and the mode of action of the synthetic TB analog, TB_KKG6K, in a drug-resistant clinical isolate of *S. aureus* and assess the peptide’s tolerance and curative potential in an *in vitro* infection model using three-dimensional human epidermis equivalents (HEEs). The results revealed a high bactericidal efficacy of TB_KKG6K at low micromolar concentrations. The peptide perturbed the bacterial cell membrane integrity by permeabilization and depolarization. TB_KKG6K showed no toxicity in the invertebrate mini-host model *Galleria mellonella* and a high level of tolerance when topically applied in HEEs. Importantly, the therapeutic potential of TB_KKG6K was confirmed in HEEs infected with *S. aureus*. The topical application of TB_KKG6K significantly reduced the bacterial load and lowered the pro-inflammatory response in the infected HEEs. These findings reinforce the antibacterial potential and therapeutic efficacy of TB_KKG6K against *S. aureus* infection, particularly in the context of a cutaneous infection.

**IMPORTANCE:**

The emergence of multidrug-resistant bacteria has rendered the exploration of novel therapeutic treatment strategies a pivotal area of research. Among the most promising candidates are amphibian-derived antimicrobial peptides (AMPs), which are ideal for the development of novel drugs due to their multifaceted mode of action. Extensive studies have been conducted on these peptides over the last decade, resulting in the development of temporin B (TB) peptide analogs that have undergone modifications to their primary sequence. These modified analogs have demonstrated enhanced antibacterial and antifungal efficacy, while exhibiting reduced hemolytic activity. TB_KKG6K has the potential to be a promising candidate for topical treatments due to its small size and high antimicrobial activity against pathogens of the human skin. In particular, it demonstrated efficacy against *Staphylococcus aureus*, a skin commensal that can become an opportunistic pathogen, causing a range of infections from minor skin infections to life-threatening diseases such as bacteremia and sepsis.

## INTRODUCTION

A significant challenge in the development of new antimicrobial drugs is the need to combat microbes that pose a severe global health threat to humans. These include the ESKAPE pathogens (*Enterococcus faecium*, *Staphylococcus aureus*, *Klebsiella pneumoniae*, *Acinetobacter baumannii*, *Pseudomonas aeruginosa*, and *Enterobacter* spp.), a group of critical multidrug-resistant bacteria (1), or drug-resistant pathogenic fungi, for instance *Candida* spp., including *Candida auris* (2). Antimicrobial peptides (AMPs) have been a subject of considerable interest as a potential new class of antimicrobial drugs and are currently a core area of research in the context of the emergence of such multidrug-resistant pathogens and the decline in the development of novel treatment strategies. These small biomolecules are secreted by organisms of various kingdoms including mammals, amphibians, invertebrates, and plants, being part of the innate immune response and representing a first defense line against invading microbes (3). A particularly promising group of AMPs is the amphibian temporins, among which temporin B (TB) is a notable example. TB was originally isolated from the mucosal glands of the European red frog *Rana temporaria* (4, 5). This small cationic peptide, comprising 13 amino acids (LLPIVGNLLKSLL), has been extensively studied for its antimicrobial activity against gram-positive bacteria.

In recent years, research has focused on the creation of synthetic TB peptide analogs with enhanced antimicrobial activity against gram-negative bacteria and reduced hemolytic activity compared to their endogenous counterparts (6–8). Several modifications were made to the primary structure of TB, resulting in the creation of the peptide analog, TB_KKG6K (amino acid sequence **KK**LLPIV**K**NLLKSLL; molecular weight 1,718.2 Daltons [Da]). TB_KKG6K displays enhanced and broadened antimicrobial activity against gram-positive and gram-negative bacteria as well as against fungal pathogens such as *Candida albicans* (9, 10). Previous studies have indicated that endogenous TB and predecessor analogs are membrane-active molecules (6, 10, 11); however, the precise mode of action remains unclear. It has been reported that many AMPs are ion-sensitive, exhibiting reduced antimicrobial activity due to structural instability and electrostatic alterations in the presence of cations, which may impede their interaction with bacterial cell membranes (12–16). A comparable susceptibility has been documented for TB_KKG6K against *C. albicans*, which may limit its systemic applicability (9). Consequently, the potential of TB_KKG6K lies in its suitability for topical treatments of mucosal or cutaneous infections.

It has been previously reported that TB and its derivatives, including TB_KKG6K, are active against *S. aureus* (6, 8, 9). While transient colonization by *S. aureus* is usually harmless, the bacterium poses a substantial risk as an opportunistic pathogen for various infections, ranging from mild cutaneous conditions to severe complications such as bacteremia, pneumonia, and endocarditis (17). The occurrence of spontaneous primary infections or the presence of preexisting lesions on the human skin can result in the development of a range of cutaneous infections, including conditions such as impetigo, abscesses, and cellulitis (18). The emergence of methicillin-resistant *S. aureus* (MRSA) strains has further complicated the situation, with the notorious USA300 strain rapidly becoming the predominant cause of community-associated MRSA skin and soft tissue infections in the United States in the 1990s and 2000s (19–21).

The choice of antimicrobial agents for the treatment of skin and soft tissue infections caused by *S. aureus* is dependent on the presentation of the disease and the antimicrobial susceptibility patterns, with particular focus on methicillin susceptibility or resistance (22, 23). *S. aureus* has the potential to cause severe conditions that may necessitate systemic therapy. However, milder, uncomplicated cases such as impetigo can be effectively treated with topical agents alone, including gentamicin, mupirocin, and retapamulin ointments (22, 24–26). There have been reports, however, on increasing resistance of *S. aureus* to some of the topical antimicrobials mentioned above (25, 27–32). A major strength of specific AMPs in comparison to antibiotics is their multifaceted mode of action, their capacity to eradicate drug-resistant bacteria, and their rapid killing of microorganisms. These qualities enable AMPs to evade the resistance mechanisms developed by microbial pathogens against currently utilized antibiotics (33–35).

The objective of this study was to investigate the efficacy and mode of action of the TB analog TB_KKG6K in *S. aureus*. To gain insight into the potential mechanism of TB_KKG6K, we examined its impact on the bacterial cell membrane and explored the possible influence on other cellular components. Furthermore, our aim was to provide a proof of concept for the applicability and curative efficacy of TB_KKG6K *in vitro*. We employed human epidermis equivalents (HEEs), which closely resemble the human epidermis, to evaluate the topical antibacterial efficacy and tolerance of TB_KKG6K *in vitro. In vivo* toxicity testing using the mini-host model *Galleria mellonella* supported the good tolerability of this antibacterial peptide.

## RESULTS

### TB_KKG6K is effective against a drug-resistant *S. aureus* isolate

**Fig 1:**
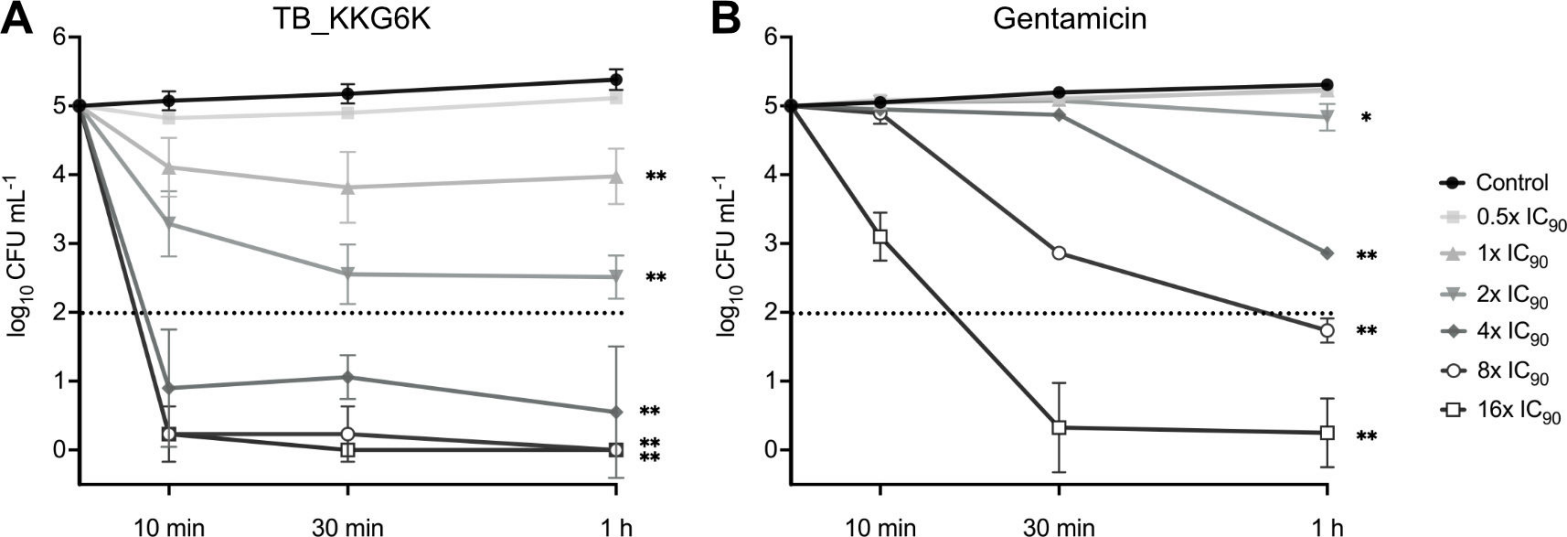
Bactericidal activity of TB_KKG6K and gentamicin. *S. aureus* cells were incubated with 0.5×, 1×, 2×, 4×, 8×, and 16× IC_90_ of (**A**) TB_KKG6K or (**B**) gentamicin for 10 min, 30 min, and 1 h at 37°C in TSB. The number of viable cells after peptide treatment was determined by plating appropriate dilutions on TSA and counting the CFU after 24 h of incubation at 37°C. The values represent the mean ± SD (*n* = 3) expressed as log_10_ CFU mL^−1^. CFU data were compared to the untreated control at the 1 h endpoint using one-way ANOVA, followed by Dunnett’s test. Significant differences between the treated cells and the control are indicated by asterisks (*P ≤ 0.05 and **P ≤ 0.005). The dotted line represents the threshold of a ≥3-log_10_ reduction in CFU mL^−1^.

The inhibitory concentration of TB_KKG6K that reduced bacterial growth by ≥90% (IC_90_) was determined in broth microdilution assay using a *S. aureus* wild-type strain and a multidrug-resistant clinical isolate. This *S. aureus* strain 195 was isolated from a patient undergoing treatment for prosthetic joint infection and deposited in the strain collection of the Research Laboratory for Biofilms and Implant Associated Infections (BIOFILM LAB), Experimental Orthopedics at the Medical University of Innsbruck, Austria. Its susceptibility profile for antibiotics was determined according to the standard protocol of EUCAST disc diffusion assay as described previously (36). As shown in Table 1, *S. aureus* 195 exhibited multiple resistance against β-lactam antibiotics. When testing TB_KKG6K, the peptide demonstrated inhibitory activity against *S. aureus* ATCC 25923 at a concentration of 4 µM. However, a concentration of 8 µM was required to achieve the same inhibitory effect against the clinical isolate *S. aureus* 195. We also assessed the susceptibility of the two *S*. *aureus* strains to the aminoglycoside antibiotic gentamicin for comparison. For this antibiotic, IC_90_ values of 0.9 and 1.8 µM for strain ATCC 25923 and strain 195 could be determined, respectively. We selected the drug-resistant clinical isolate of *S. aureus* as a test organism for further investigations. To ascertain the bactericidal efficacy of TB_KKG6K, a colony-forming unit (CFU) plating assay was conducted, and the killing kinetics were examined. *S. aureus* cells were inoculated in tryptic soy broth (TSB) and exposed to 0.5×–16× IC_90_ of TB_KKG6K for 10 min, 30 min, and 1 h, or left untreated (negative control). We compared the antibacterial potential of the peptide with that of gentamicin under the same assay conditions. At each incubation time point, the appropriate dilutions were plated on tryptic soy agar (TSA) and, after a further incubation for 24 h, the number of viable cells, represented by CFUs, was determined. The colony counts were then compared to those of the untreated control (Fig. 1). The treatment with TB_KKG6K at concentrations corresponding to 1×–16× IC_90_ resulted in a significant reduction of CFU counts (Fig. 1A). At concentrations corresponding to 4×–16× IC_90_, however, a bactericidal activity was detectable already within 10 min of exposure, evidenced by the reduction in CFU of ≥3-log_10_. In comparison, gentamicin also demonstrated a concentration and time-dependent killing dynamic. This was, however, not as fast and effective as with TB_KKG6K. A significant reduction in CFUs could be detected with concentrations ≥2× IC_90_ at 1 h after exposure to the antibiotic, whereby a bactericidal effect could only be observed with 8× and 16× IC_90_ (Fig. 1B).

**TABLE 1:** Antibiogram of the clinical isolate *S. aureus* 195

Antibiotic	Concentration^*a*^	Susceptibility
Amikacin	30	S
Aminopenicillin	10	R
Aminopen + clavulanic acid	20/10	S
Azithromycin	15	S
Aztreonam	30	R
Cefazolin	30	S
Cefepime	30	S
Cefotaxime	30	S
Cefoxitin	30	S
Ceftazidime	30	R
Ceftriaxone	30	S
Cefuroxime	30	S
Ciprofloxacin	5	S
Clindamycin	2	S
Doripenem	10	S
Ertapenem	10	S
Fosfomycin	200	S
Fusidic acid	10	S
Gentamicin	10	S
Imipenem	10	S
Levofloxacin	5	S
Linezolid	30	S
Meropenem	10	S
Moxifloxacin	5	S
Penicillin G	1	R
Piperacillin-tazobactam	100/10	S
Rifampicin	5	S
Tetracycline	30	S
Tigecycline	15	S
Trimethoprim + sulfonamide	5	S
Vancomycin	5	S

^*a*^ Concentration tested (μg per disc) (36); R, resistant; S, susceptible.

### TB_KKG6K affects the bacterial cell membrane integrity

In order to gain insight into the mode of action of TB_KKG6K, the effect of the peptide on the bacterial membrane was investigated. We determined its potential to disrupt the membrane polarity using the fluorescent dye 3,3′-dipropylthiadicarbocyanine iodide [DiSC_3_(5)]. This voltage-sensitive dye dimerizes in intact cell membranes, thereby quenching the fluorescence signal. Following depolarization, the dye is released into the supernatant, resulting in a detectable increase in fluorescence (37).

**Fig 2:**
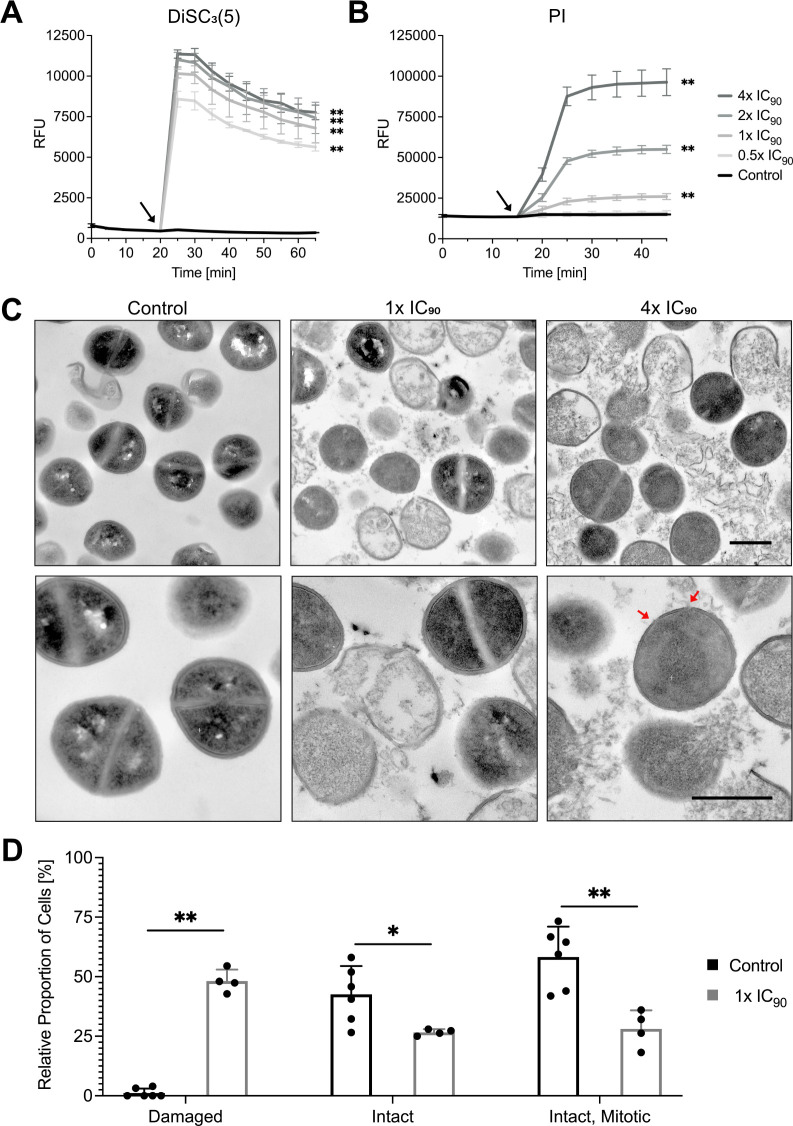
Membrane activity of TB_KKG6K. (**A**) The membrane depolarization activity was detected with the fluorescent dye DiSC_3_(5). (**B**) The membrane permeabilization activity was identified by applying the fluorescent dye PI. *S. aureus* was exposed to TB_KKG6K at concentrations corresponding to 0.5×, 1×, 2×, and 4× IC_90_. Controls remained untreated. The fluorescence signals are depicted as relative fluorescence units (RFU). The RFU values were calculated by subtracting the background fluorescence of 4-(2-hydroxyethyl)-1-piperazineethanesulfonic acid (HEPES) buffer (**A**) or phosphate-buffered saline (PBS; **B**) with or without TB_KKG6K in the absence of cells (negative control). Values represent mean ± SD (*n* = 3). Significant differences between the data of the peptide-treated samples and the untreated control are indicated by an asterisk (**P ≤ 0.005). Arrows (black) indicate the time point of peptide addition. (**C**) Transmission electron microscopy was performed to examine the effects of TB_KKG6K on the cellular morphology of *S. aureus*. Bacterial cells (4 × 10^8^ CFU) were treated with either 1× IC_90_ or 4× IC_90_ of TB_KKG6K for 1 h at 37°C, and an untreated control was included. The upper panel depicts images taken with 6,300× magnification, and the lower panel presents a more detailed view of the cells at 12,500× magnification. Arrows (red) indicate pore-like structures. Scale bars, 500 nm. (**D**) The number of intact, mitotic or damaged *S. aureus* cells was quantified in representative transmission electron microscopy images of the untreated control (*n* = 6) and the samples treated with 1× IC_90_ of TB_KKG6K (*n* = 4). The results are presented as a percentage of the total cell count per image. Significant differences between the treated samples and the untreated control are indicated by asterisks (*P ≤ 0.05 and **P ≤ 0.005).

TB_KKG6K induced a rapid depolarization of the *S. aureus* membrane potential (Fig. 2A), which was evidenced from a time and peptide concentration-dependent increase in fluorescence intensity. No change in the signal intensity could be observed in the untreated *S. aureus* cells.

Next, the potential of TB_KKG6K to permeabilize the bacterial plasma membrane was investigated. To this end, the membrane impermeant dye propidium iodide (PI) was employed, which intercalates with the DNA of cells with impaired plasma membrane, leading to a detectable increase in fluorescence (38). TB_KKG6K was applied at concentrations corresponding to the 0.5×, 1×, 2×, and 4× IC_90_ in *S. aureus* after the addition of the PI stain to the cells, and the resulting fluorescent signal was recorded. The addition of the peptide to the cells resulted in rapid membrane permeabilization, which was concentration and time dependent (Fig. 2B). It is noteworthy that the permeabilization of cells reached a saturation point after 10 min post-peptide addition.

The impact of TB_KKG6K on the membrane integrity was also evident from the morphological changes of the treated bacterial cells, which were visualized by transmission electron microscopy (TEM). The images demonstrated that the untreated *S. aureus* cells exhibited an intact structure of the cell wall and membrane. The cellular cytoplasm appeared electron-dense, indicative of a high ribosome content (Fig. 2C) (39, 40). In contrast, signs of membrane disruption were evident in the TB_KKG6K-treated cells. The formation of pore-like structures in the outer layers of the bacterial cells, the damage to the cell membrane, the decomposition of intracellular structures, and cell lysis were discerned at a concentration equivalent to the 1× IC_90_. These defects were further intensified at 4× IC_90_, resulting in an augmented number of lysed bacterial cells and intracellular content that aggregated extracellularly (Fig. 2C). A quantitative analysis of representative images was conducted, wherein cells were assessed for integrity, damage, and mitotic status. The results indicate that at 1× IC_90_, approximately half of the cells were visibly damaged, and the amount of intact cells and cells that were undergoing mitosis was reduced in comparison to the untreated control (Fig. 2D).

### TB_KKG6K shows no toxicity in an invertebrate model

**Fig 3:**
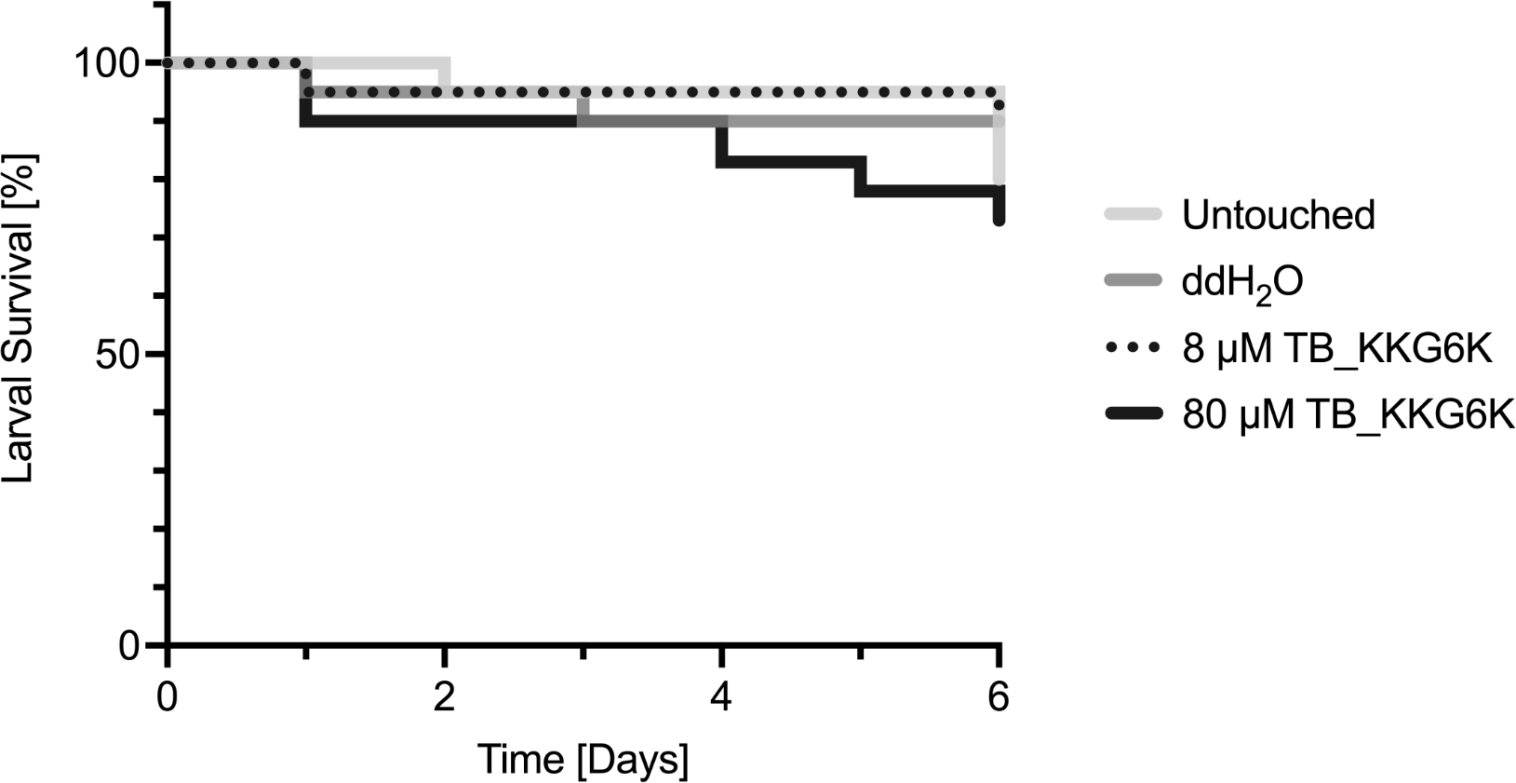
Toxicity assessment of TB_KKG6K in *G. mellonella*. Survival of *G. mellonella* larvae after injection with 20 µL of either 8 µM (0.785 mg kg^−1^) or 80 µM (7.85 mg kg^−1^) of TB_KKG6K in comparison with the untouched and ddH_2_O-treated control. The survival is statistically not different. For larvae treated with 80 µM vs. untouched: *P* = 0.92 (log rank [Mantel-Cox] test), and *P* = 0.85 (Gehan-Breslow-Wilcoxon test). For larvae treated with 80 µM vs. ddH_2_O: *P* = 0.27 (log rank [Mantel-Cox] test), and *P* = 0.30 (Gehan-Breslow-Wilcoxon test) (*n* = 20).

The potential of TB_KKG6K to serve as a candidate molecule for drug development was evaluated by assessing its *in vivo* toxicity using the invertebrate mini-host *G. mellonella*. The larvae of *G. mellonella* were injected with two concentrations of the peptide, 8 and 80 µM prepared in sterile distilled water (ddH_2_O), corresponding to 1× IC_90_ and 10× IC_90_, respectively. Larvae left untouched and larvae injected with ddH_2_O served as controls. The survival analysis revealed that larvae treated with 8 µM of TB_KKG6K survived as well as the ddH_2_O control. The administration of the peptide at 80 µM resulted in a mild, albeit insignificant reduction in the survival rate, which became evident from day 4 post-treatment onwards (Fig. 3).

### TB_KKG6K is well tolerated in HEEs

To assess the potential of TB_KKG6K to be applied as a topical drug against bacterial skin infection in a more complex context, three-dimensional (3D) reconstructed human skin models were used to test first the peptide’s tolerability and subsequently its curative effect. To address this objective, commercially available HEEs cultivated for 9 days at an air-liquid interface (ALI 9) were used. We tested TB_KKG6K at a concentration of 1 mM, which corresponds to 125× of IC_90_, previously determined in broth microdilution assay, to show the good tolerability of the peptide at a high concentration that may also effectively reduce the bacterial colonization of the models. The cytotoxicity of TB_KKG6K was examined using a colorimetric skin irritation assay based on 3-[4,5-dimethylthiazol-2-yl]-2,5-diphenyltetrazolium bromide (MTT). The metabolic activity of the peptide-treated HEEs was calculated as the percentage relative to the negative control, which was treated with Dulbecco’s phosphate-buffered saline (D-PBS) and assigned a metabolic activity of 100%. The positive control, namely 5% (wt/vol) sodium dodecyl sulfate (SDS) applied in 30 µL, resulted in the significant reduction of metabolic activity to 0.9% ± 0.1% (**P ≤ 0.005), thereby confirming the categorization of SDS as an irritant. In contrast, the application of 52 µg peptide in 30 µL ddH_2_O did not affect the metabolic activity of the cells in the HEEs. The metabolic activity reached 96.5% ± 8.4%. This value is well above the irritant threshold of 50% metabolic activity as defined by the Organization for Economic Cooperation and Development (OECD) guidelines (41). This result confirmed that TB_KKG6K can be categorized as a non-irritant compound even when applied at a concentration corresponding to 125× IC_90_.

**Fig 4:**
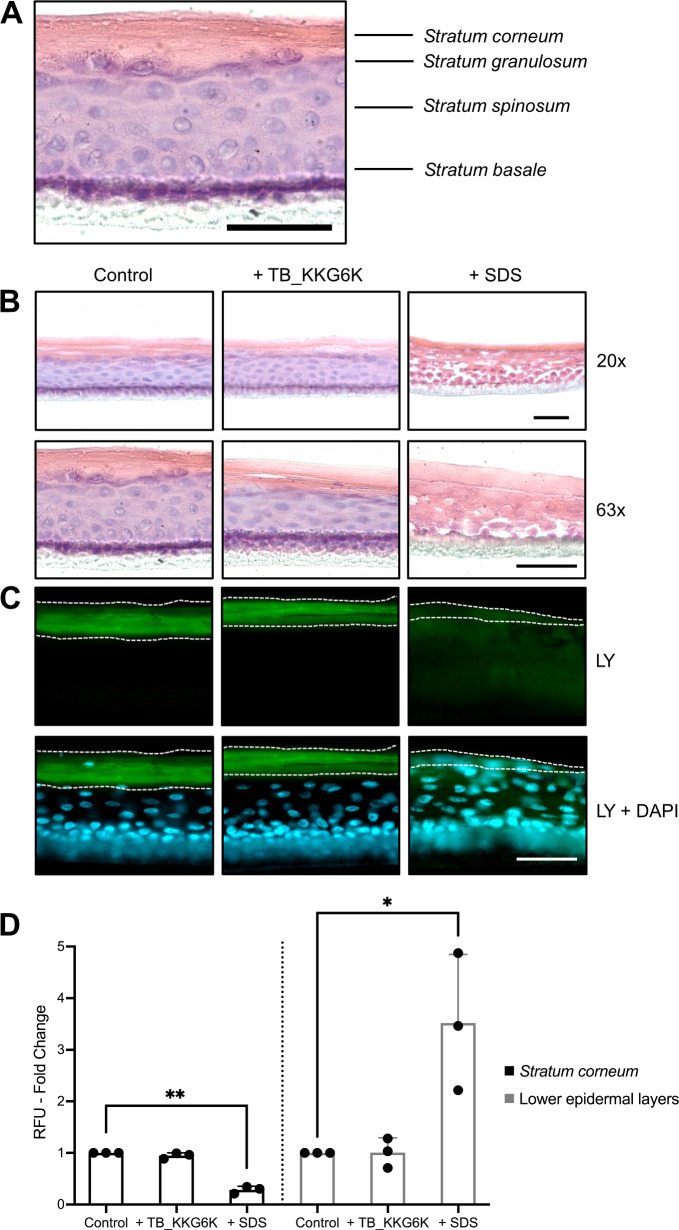
Tolerance of TB_KKG6K in HEEs. HEEs were treated with 18 µL of TB_KKG6K (1.7 mg mL^−1^ ddH_2_O) at ALI 9 for 24 h or 1% (wt/vol) SDS solution at ALI 10 for 1 h, after which the effects on the model were analyzed in comparison to a negative HEE control (ALI 10) by histochemistry. (A and B) Cryo-sections were stained with H&E. The images were captured at two magnifications: 20× and 63×. Panel A shows the magnified image of the “control” in panel B at magnification 63× and depicts the different layers of the reconstructed HEE. (**C**) The application of 50 µL LY (1 mg mL^−1^) for 2 h at 32°C with 5% CO_2_ was conducted at ALI 10. The cryo-sections were mounted in Fluoroshield with DAPI (blue). Dotted lines delineate the stratum corneum. All images were taken with the same exposure time. Scale bars, 50 µm. (**D**) The signal intensity of fluorescence emitted from LY was quantified in the stratum corneum and the lower epidermal layers of HEEs at ALI 10. The difference in intensity is depicted as fold change in RFU in comparison to the negative control. Values represent the mean ± SD (*n* = 3). Significant differences between the treated samples and the negative control are indicated by asterisks (*P ≤ 0.05 and **P ≤ 0.005).

The tolerability of TB_KKG6K was further underscored by the intact structure of the epidermal layers of the HEE model that had been exposed to the peptide. HEEs (ALI 9) were treated with 18 µL of a TB_KKG6K solution in ddH_2_O (1.7 mg mL^−1^), corresponding to 31 µg total amount of TB_KKG6K per model for 24 h. At ALI 10, the models were harvested, and sections were prepared and stained with hematoxylin and eosin (H&E). A control was included that was treated with 1% SDS (wt/vol) for 1 h, as SDS is known to disrupt the skin barrier (42). Analysis of the negative control revealed the presence of the distinct layers of the human epidermis, consisting of the stratum corneum, stratum granulosum, stratum spinosum*,* and stratum basale (Fig. 4A). Application of TB_KKG6K resulted in the HEEs retaining their integrity, as no evident changes in the morphology and thickness of the skin layers were observed compared to the untreated control (Fig. 4B). In contrast, the epidermal layers of the HEEs were subjected to significant damage by the detergent, resulting in notable alterations to their morphology and structure. The cell membranes of the keratinocytes appeared to be compromised, and the distinct cellular architecture was lost (Fig. 4B).

We further analyzed the impact of TB_KKG6K on the membrane permeability barrier of the HEEs. The application of the membrane permeability barrier marker Lucifer Yellow (LY) revealed the accumulation of the dye in the stratum corneum of both the TB_KKG6K-treated HEEs and the negative control (Fig. 4C), indicating that the outside-in permeability barrier was not compromised by this treatment. In contrast, SDS-exposed HEEs exhibited increased membrane permeability, which was evidenced by LY penetration of the stratum corneum into the deeper epidermal layers. These microscopic observations were also reflected in the quantification of the LY-specific fluorescence signal intensity distributed in the stratum corneum compared to that in the lower epidermal layers in Fig. 4D.

**Fig 5:**
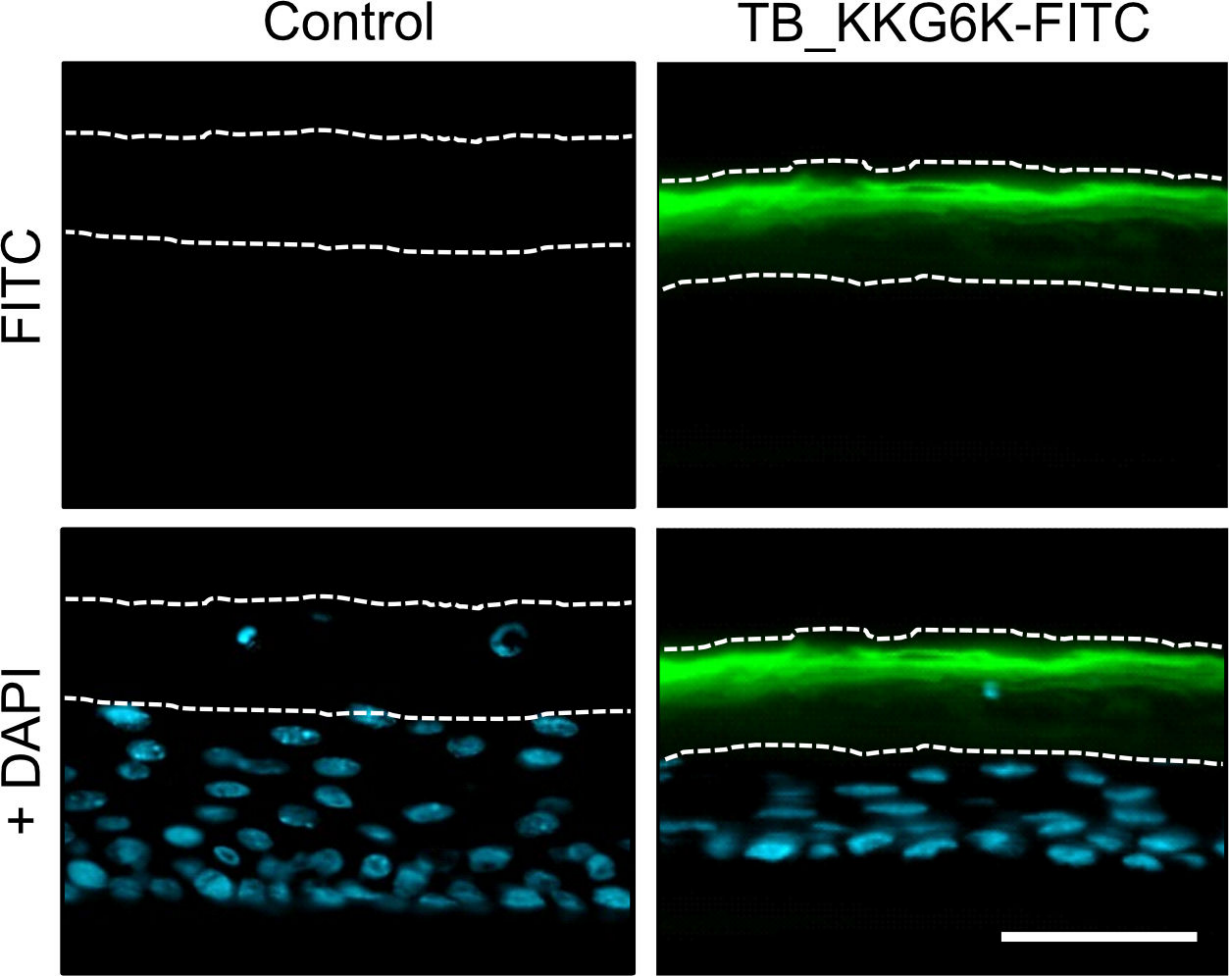
Penetration depth of TB_KKG6K. Thirty-one µg of FITC (green) labeled TB_KK6GK in 18 µL ddH_2_O was applied to HEEs for 24 h at ALI 9. The sections were mounted in Fluoroshield with DAPI (blue). Dotted lines delineate the stratum corneum. All images were taken with the same exposure time. Scale bar, 50 µm.

We also aimed to analyze the penetration ability of TB_KKG6K in the HEEs. To reach this goal, we topically applied 1 mM fluorescein isothiocyanate (FITC)-labeled TB_KKG6K (TB_KKG6K-FITC) and incubated the models for 24 h. The results showed that the peptide only penetrated the stratum corneum but did not reach the lower epidermal layers (Fig. 5).

### TB_KKG6K exhibits therapeutic effects in HEEs infected with *S. aureus*

In order to assess the curative potential of TB_KKG6K against cutaneous infections caused by *S. aureus*, a simple infection model was established in HEEs. The HEEs were inoculated with approximately 3,000 CFU of *S. aureus* at ALI 8 and cultivated for 24 h (ALI 9) and 48 h (ALI 10) at 32°C and 5% CO_2_. For the treatment, 31 µg of TB_KKG6K in 18 µL ddH_2_O was applied 24 h after infection at ALI 9, and the HEEs were further incubated for another 24 h before they were analyzed at ALI 10. A 24-h treatment with TB_KKG6K was chosen to assess the peptide’s potential to sustainably reduce the bacterial load and inhibit regrowth of cells that may have survived the treatment. Controls were included for each experiment consisting of uninfected, untreated HEEs and infected, untreated HEEs.

**Fig 6:**
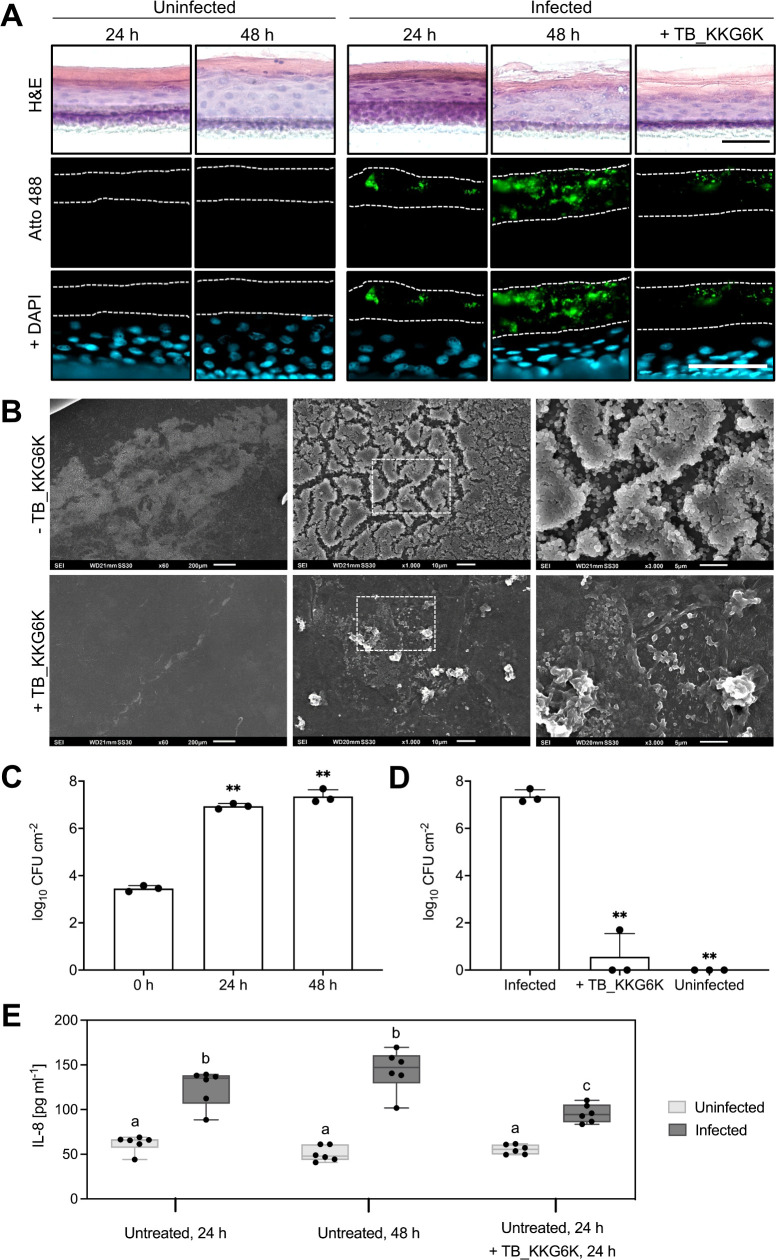
*S. aureus* infection and TB_KKG6K treatment in HEEs. (**A**) HEEs were infected with 3,000 CFU of *S. aureus* at ALI 8 and the effects were histologically analyzed after 24 h (ALI 9) and 48 h (ALI 10) of infection. In total, 31 µg of TB_KKG6K in 18 µL ddH_2_O was applied 24 h p.i. The infected, untreated, and TB_KKG6K-treated HEEs were compared to uninfected, untreated controls. Cryo-sections were stained with H&E, and *S. aureus* cells (green) were visualized in the cryo-sections by immunofluorescence staining using the primary anti-*S*. *aureus* antibody and an anti-rabbit IgG-Atto 488 secondary antibody. Nuclei were counterstained by mounting with Fluoroshield with DAPI (blue). Dotted lines delineate the stratum corneum. All images depicting fluorescent signals were taken with the same exposure time. Scale bars, 50 µm. (**B**) The infected, untreated (−TB_KKG6K) and treated (+TB_KKG6K) HEEs were harvested at ALI 10 and analyzed with scanning electron microscopy. The images were captured at three different magnifications: 60×, 1,000×, and 3,000× (from left to right). The dotted lines indicate the sector that is further magnified. (C and D) HEEs were homogenized, and appropriate dilutions were plated onto TSA plates to determine the CFU load per model (0.6 cm^2^). (**C**) The dynamic increase of CFUs compared to the inoculum over 24 h (ALI 9) and 48 h (ALI 10) of infection. (**D**) The significant reduction in CFUs after TB_KKG6K treatment compared to the infected, untreated control, and uninfected control (ALI 10). The values in panels **C** and **D** represent the mean ± SD (*n* = 3) expressed as log_10_ CFU cm^−2^. Significant differences between the data of treated samples and the untreated control at respective time points are indicated by asterisks (**P ≤ 0.005). (**E**) Boxplot depicting IL-8 levels in CCM from uninfected and infected HEEs. Data represent technical duplicates from three independent experiments (*n* = 3). Statistical analysis was performed using a two-way ANOVA followed by Tukey’s range test for group comparisons. Significant differences between groups are shown using a compact letter display, where different letters represent *P* ≤ 0.05.

H&E staining results indicated that the bacteria did not drastically affect the tissue structure visually within the first 24 h of infection, while a significant disruption of the upper layer of the stratum corneum was evidently observed after 48 h of infection in comparison to the uninfected control. The tissue damage observed with H&E staining in HEEs infected for 48 h could be mitigated by the administration of TB_KKG6K at 24 h post-infection (p.i.) at ALI 9 (Fig. 6A, upper panel). As this staining does not permit visualization of *S. aureus* cells within the tissue, we proceeded to detect the bacteria by immunofluorescent staining with a *S. aureus*-specific antibody. We observed that at 24 h p.i., the bacteria predominantly colonized the surface of the stratum corneum, followed by a strong proliferation on the stratum corneum with a few bacteria also reaching the deeper layers of the epidermis after another 24 h of incubation (Fig. 6A). When TB_KKG6K was applied 24 h p.i., the extensive colonization of the stratum corneum with bacteria and their penetration of the HEEs could be effectively prevented 48 h p.i. (Fig. 6A).

The potential of TB_KKG6K to inhibit the formation of bacterial biofilms and to mitigate bacterial infection in HEEs was further evidenced with scanning electron microscopy. The microscopic images demonstrate the presence of a bacterial biofilm on the surface of infected HEEs that were left untreated (Fig. 6B, upper panels). The administration of TB_KKG6K effectively prevented the formation of an extensive biofilm, and the remaining bacterial cells exhibited notable morphological changes, becoming smaller and deformed (Fig. 6B, lower panels).

These observations are well reflected in the determination of total bacterial load. Although *S. aureus* cells exhibited a high rate of multiplication within the first 24 h of infection, TB_KKG6K treatment demonstrated the capacity to not only prevent the growth of new bacteria but also to kill the bacteria, resulting in a near-total elimination of viable CFUs at ALI 10 (Fig. 6C and D).

Notably, when analyzing the penetration depth of TB_KKG6K-FITC in infected HEEs, we could not observe any difference compared to that in intact HEEs (data not shown). This result suggests that the infection does not render the stratum corneum more permeable for this peptide under the applied test conditions.

Finally, the release of the pro-inflammatory cytokines IL-1α, IL-6, and IL-8 into the conditioned culture medium (CCM) in HEEs infected with *S. aureus* was assessed in response to TB_KKG6K treatment. For comparison, uninfected, untreated controls, and infected, untreated controls were included. While no secretion of IL-1α and IL-6 could be detected in the CCM under any of the conditions tested, the level of secreted IL-8 in uninfected HEEs in response to TB_KKG6K could be assessed. The concentration of IL-8 remained unchanged compared to the uninfected and untreated HEEs (Fig. 6E). This further underlined the compound’s good tolerability. However, the concentration of IL-8 in HEEs was increased after 24 h of infection with *S. aureus* and further augmented after 48 h in comparison to the uninfected, untreated HEEs, indicating a pro-inflammatory response of the keratinocytes upon bacterial infection. Importantly, TB_KKG6K treatment significantly reduced the secretion of IL-8 in infected HEEs in comparison to the infected, untreated control (Fig. 6E).

## DISCUSSION

This study provides for the first time detailed insight into the antibacterial activity of the TB analog TB_KKG6K and demonstrates its curative potential against the human skin pathogen *S. aureus*.

Our data showed that TB_KKG6K exhibited bactericidal activity at low micromolar concentrations. The antibacterial efficacy was dependent on the concentration of TB_KKG6K and the duration of peptide exposure. The peptide seemed to act more effectively than gentamicin, because it rapidly killed the cells of the *S. aureus* clinical isolate at concentrations close to its IC_90_, reaching a ≥3-log_10_ reduction in CFU mL^−1^ within minutes after exposure to concentrations ≥4× IC_90_.

The antibacterial efficacy of TB_KKG6K is closely linked to its membrane perturbation activity. TB_KKG6K impaired the bacterial membrane integrity by inducing membrane depolarization and permeabilization in a concentration- and time-dependent manner. Rapid depolarization was observed right after exposure of the bacterial cells to the peptide, even at a subinhibitory concentration, whereas membrane permeabilization required concentrations ≥1× IC_90_.

To date, the mode of interaction of TB and TB analogs like TB_KKG6A and TB_KKG6K with membranes has been mainly studied with gram-negative bacteria (5). The capacity of TB to interact with biomembranes has previously been investigated using model lipid bilayers that mimic the membranes of gram-negative and gram-positive bacteria. These studies demonstrated that TB can cause lipid segregation, peptide aggregation, and formation of protrusions within artificial cell membranes (5, 43–45). Similarly, TB_KKG6K was shown to induce leakage in large unilamellar vesicles (LUVs) composed of specific lipids that are found predominantly in cell membranes of gram-negative and gram-positive bacteria, respectively. However, LUVs composed of anionic phospholipids, such as phosphatidylglycerol, which mimic the membrane of gram-positive bacteria, were more sensitive to peptide-induced leakage than those LUVs containing less-anionic lipids found in the membranes of gram-negative bacteria (10).

Due to the rapid onset of cell membrane depolarization and permeabilization, it is not possible to discriminate which of these events occurs first. It can be assumed that the two effects are interdependent and exert a dynamic influence on the bacterial membrane. It has been reported that the extent of the membrane effects and the degree of cell killing induced by AMPs may vary considerably. For instance, rapid depolarization of the bacterial membrane can be observed at sub-inhibitory peptide concentrations, whereas rapid bacterial killing can be induced even by AMPs that have minimal depolarization and permeabilization potential (34, 46–48).

However, in our study, a considerable membrane depolarization could already be observed at a sub-inhibitory concentration (0.5× IC_90_), similar to that at the bactericidal concentration of 4× IC_90_. This lets us assume that the interaction of the peptide with the bacterial cells induced membrane depolarization, which does not necessarily lead to cell membrane permeabilization and subsequent bacterial cell death. Cells can survive this impact as long as the membrane function can be restored. Our assumption was supported by the observation that at 0.5× IC_90_, the increase of the PI signal and the reduction in CFU were not significantly different from those of the untreated control, although a strong membrane depolarization could be observed. A significant increase in PI signal coincided with a substantial decrease in CFU at peptide concentrations ≥1× IC_90_, reflecting the negative effect of TB_KKG6K on cell membrane integrity and explaining the cell killing potential, which culminated in ≥3-log_10_ reduction of the CFU at peptide concentrations ≥4× IC_90_.

The TEM data analysis indicated that exposure of *S. aureus* to TB_KKG6K resulted in growth arrest, leakage of the bacterial cell wall and membrane, the disintegration of the subcellular structures, and the extracellular aggregation of cell debris, thereby supporting the aforementioned scenario.

Prior to obtaining clinical approval, drug candidates must undergo intensive testing to guarantee their safe utilization. The typical approach is to translate the findings from *in vitro* cell culture to animal testing. We wanted to consider the 3Rs (replace, reduce, and refine) and the necessity of proving results in human-based models in the initial phase of drug screening (49). By using HEEs, we have taken the first steps toward the evaluation of the topical applicability and therapeutic potential of TB_KKG6K. HEEs are relatively simple epidermis models that are constructed from primary keratinocytes and mimic the intricate structure of human skin, thereby closely resembling the microenvironment of the affected host. However, they do not fully replicate the structure and function of natural skin. This is due to the absence of other cell types that are resident in the skin, such as fibroblasts and immune cells. However, HEEs may serve as optimal *in vitro* models for the testing of substances that still require optimization to improve their efficacy and allow studies to be conducted under standardized conditions and with high reproducibility compared to *ex vivo* human and animal skin (50, 51). The utilization of HEEs represents an approach that facilitates an efficient and cost-effective transition from *in vitro* to *in vivo* pre-clinical testing, which is a mandatory step in the successful development of novel therapeutics and supports the efforts to consider the 3Rs in the laboratory. As evidenced by our previous research, the utilization of 3D skin models has emerged as a valuable tool for investigating the applicability of AMPs (9, 52).

It should be noted that the bactericidal effects of TB_KKG6K observed *in vitro* may not fully account for the complexity of bacterial responses during infection. A crucial point to consider is the phenomenon of persister cells, which exhibit metabolic inactivity and a dormant state. This enables them to withstand the treatment with antibacterial drugs without developing genetic resistance. They resume growth once drug concentrations decline to sub-lethal levels (53). Furthermore, *S. aureus* may evade AMP-mediated killing by modifying its cell membrane, sequestering peptides, or secreting proteins such as proteases (54, 55). Consequently, it is advisable to utilize concentrations that exceed the minimal bactericidal concentration in order to achieve rapid and sustained suppression of bacterial growth and prevent the development of resistance, provided that the applied concentration is well tolerated.

Given that HEEs exhibit increased permeability due to differences in the composition of the lipid content in comparison to that of natural skin, these epidermis models have been shown to be more sensitive to administered compounds (51). To demonstrate the tolerability of the peptide in HEEs and its potential efficacy as a therapeutic agent against *S. aureus* infection, we selected the highest feasible concentration for the application of TB_KKG6K, namely 125 x IC_90_. Indeed, the use of TB_KKG6K at this high concentration did not result in any irritation of the epidermal layers or alterations to the histological structure or impairment of the outside-in permeability barrier. This finding corroborates the results of an earlier study that we conducted with an epidermis model (Phenion OS-REp) and a full-thickness skin model that had been exposed to a comparable amount of AMPs (9, 52). Therefore, our study provides evidence of the consistency and reproducibility of results across different reconstructed human epidermis models. Additionally, the peptide demonstrated favorable tolerability *in vivo*, as evidenced by the comparable vitality of *G. mellonella* larvae injected with the peptide to that of the controls. The use of the mini-host model, *G. mellonella* larvae, in the early stages of drug safety and toxicity testing *in vivo* has been demonstrated to be a reliable predictor of results in mammals and offers a financially attractive and ethically acceptable alternative to rodent testing (56–58).

A proof of principle experiment should demonstrate the therapeutic potential of TB_KKG6K in our study. To this end, HEEs were selected as a preliminary model for establishing a cutaneous bacterial infection, and the infected HEEs were treated with the peptide.

Despite the simplicity of HEEs compared to human skin, their use offers a valuable and insightful approach when screening compounds like TB_KKG6K for their anti-infective efficacy and potential for future drug development. We could show that *S. aureus* colonized the stratum corneum, but also penetrated deeper into the HEE layers, which reflected the bacterial growth dynamics reported in other studies (59, 60). The colonization resulted in the formation of a substantial bacterial biofilm on the HEE surface. Biofilms form on biotic and abiotic surfaces by the adhesion of bacterial cells that secrete extracellular polymeric substances. Staphylococcal biofilm is known to contribute to the pathogenesis of non-healing skin infections (61), as it protects the embedded bacteria from the human immune response and reduces their susceptibility to antibiotics (62). Thus, the establishment of an infection of the HEEs with *S. aureus* was reflected by the increase of CFU numbers, which correlated with the elevation of the concentration of secreted IL-8. The production of pro-inflammatory cytokine IL-8 has been found to be induced in keratinocytes upon infection, where it serves as a chemotactic factor that recruits immune cells, like neutrophils, to the site of IL-8 secretion and promotes keratinocyte proliferation, contributing to wound healing. Furthermore, it processes inflammatory response cascades (63, 64). Our observation of increased IL-8 secretion upon *S. aureus* infection in HEEs is consistent with numerous studies that have reported the induction of IL-8 by *S. aureus* infection, for example in cultured keratinocyte monolayers and *ex vivo* models of human skin tissue (65, 66). In addition, IL-8 has been identified to be the predominant inflammatory signal in response to *S. aureus* colonization of the skin (66). The inability to detect other cytokines, like IL-1α or IL-6, that are known to be produced by keratinocytes in response to bacterial infection may be attributable to several, not further investigated factors. However, the use of epiCS HEEs, which are notably small in size, combined with the collection of CCM in 24-h intervals for subsequent analysis, could be one reason for cytokine concentrations that were below the detection level.

Importantly, TB_KKG6K reduced biofilm formation, killed *S. aureus* cells, and prevented negative effects evoked by the bacterial colonization of the HEEs, like the disruption and exfoliation of the stratum corneum. This is remarkable considering that TB_KKG6K accumulated in the *s*tratum corneum and further underlines the good tolerability and curative potential of this peptide. In addition, the reduction in the bacterial load in response to TB_KKG6K treatment was also reflected by the measurement of a decreased level of IL-8 in the CCM of infected HEEs. It is plausible that the reduction in IL-8 production may have also contributed to the diminished tissue damage observed in the histological analysis. These findings are consistent with those of previous studies that have demonstrated anti-inflammatory effects associated with TB and TB_KKG6A treatment in both *in vitro* and *in vivo* models (7, 58, 67–69).

Despite its small size (1.7 kDa), TB_KKG6K may still be too large to allow sufficient skin penetration to reach deeper layers, as the stratum corneum has a reduced permeability to aqueous solutions and compounds with a molecular mass greater than 500 Da (70, 71). It has to be investigated in more detail in future experiments if TB_KKG6K can reach deeper layers of the skin when the stratum corneum is compromised, for example, by wounding or inflammatory skin conditions, like psoriasis or atopic dermatitis. We could, however, not detect any difference in the penetration depth of TB_KKG6K in intact and infected HEEs, respectively. Apart from the ability to penetrate the skin, TB_KKG6K has been reported to be susceptible to proteolysis (9). These limitations must be considered when developing AMPs, such as TB_KKG6K, for the treatment of cutaneous infections. They could be overcome by combining the peptides with an appropriate formulation. Smart drug delivery systems, such as nanogels, have been shown to promise sufficient stability, optimal tissue penetration, and delivery of AMPs and compounds to the site of infection (72, 73). Further studies will need to focus on improving these factors, while also maintaining the tolerability of the AMPs and avoiding potential adverse effects in the patient, such as hemolysis or allergic reactions.

In conclusion, we propose that TB_KKG6K has the ability to cure bacterial infections and has considerable potential as a therapeutic agent for the treatment of *S. aureus* infections of human skin. TB_KKG6K could be applied as a single compound or in combination with licensed drugs to treat MRSA strains of *S. aureus*, where other therapeutic options are ineffective. Further rational design to improve species specificity and delivery to the site of infection will facilitate the development of tailored antimicrobial treatment strategies for patients suffering from cutaneous bacterial infections.

## MATERIALS AND METHODS

### Peptide synthesis

TB_KKG6K and the FITC-conjugated peptide (TB_KKG6K-FITC) were synthesized and purified by reversed-phase high-performance liquid chromatography (RP-HPLC) as described previously (10).

### Strains, media, and cultivation conditions

*S. aureus* (ATCC 25923) was obtained from the American Type Culture Collection (ATCC, Manassas, VA, USA). The clinical strain *S. aureus* 195 was isolated from patients undergoing treatment for prosthetic joint infection. Its susceptibility profile for antibiotics was determined according to the standard protocol of EUCAST disc diffusion assay as described previously (36). Growth media and solutions used in this study are detailed in Table 2. For the cultivation of *S. aureus*, single colonies grown on TSA were used to inoculate 5 mL of TSB. Following overnight incubation (o/n) at 37°C with shaking at 200 rpm, a 1:100 dilution in TSB was prepared to allow exponential bacterial growth until the culture reached an optical density (OD) of 0.5 at 620 nm (OD_620_), which corresponded to 1 × 10^8^ CFU mL^−1^.

**TABLE 2:** Media and solutions used in this study

Media/solution	Composition^*a*^
Tryptic soy broth (TSB)	1.7% casein peptone, 0.3% soy peptone, 0.5% NaCl, 0.25% K_2_HPO_4_, 0.25% d-(+)-glucose
Tryptic soy agar (TSA)	Tryptic soy broth, 2% agar
Sabouraud agar (SBA)	1% peptone, 4% d-(+)-glucose, 2% agar, pH 5.6
Lysogeny broth (LB) agar	1% NaCl, 1% peptone, 0.5% yeast extract, 2% agar
Phosphate-buffered saline (PBS)	0.05% KH_2_PO_4_, 0.28% K_2_HPO_4_, 0.9% NaCl, pH 7.4
Dulbecco’s phosphate-buffered saline (D-PBS)	0.02% KCl, 0.02% KH_2_PO_4_, 0.8% NaCl, 0.115% Na_2_HPO_4_, pH 7.0

^*a*^ Percent values are depicted as wt/vol.

### Broth microdilution assays

The IC_90_ was determined for microorganisms using broth microdilution assays performed in 96-well microtiter plates (Nunclon Delta, Thermo Fisher Scientific, Waltham, MA, USA). A total of 100 μL of *S. aureus* (1 × 10^4^ cells mL^−1^ in TSB) was mixed with 100 µL of serially diluted twofold concentrated TB_KKG6K or gentamicin (Sigma-Aldrich) in TSB and incubated at 37°C for 24 h under static conditions. After incubation, the microorganisms were resuspended by vigorous pipetting, and the OD_620_ was measured with a multimode microplate reader (CLARIOstar Plus, BMG Labtech, Ortenberg, Germany). The OD_620_ of the untreated control was set as 100% growth. All samples were prepared in technical triplicates, and the assays were repeated at least three times.

### Time-kill studies of TB_KKG6K

*S. aureus* cells were diluted in TSB to achieve a concentration of 1 × 10^5^ CFU mL^−1^. A total of 100 µL of the culture was pipetted into 2 mL tubes. Subsequently, 100 µL of TSB (untreated control) or 100 µL of twofold concentrated TB_KKG6K or gentamicin, resulting in final concentrations corresponding to 0.5×, 1×, 2×, 4×, 8×, and 16× IC_90_, were added. All conditions were prepared in technical duplicates and incubated at 37°C with continuous shaking at 200 rpm. At 10 min, 30 min and 1 h aliquots were taken and plated in appropriate dilutions onto TSA plates in duplicates to count the viable cells after incubation at 37°C for 24 h. The experiments were performed at least three times.

### Membrane depolarization assay

The membrane depolarization potential of TB_KKG6K in *S. aureus* was evaluated by a DiSC_3_(5) assay. Bacterial cells were washed three times in 5 mM HEPES supplemented with 20 mM glucose, 100 mM KCl (pH 7.4). Subsequently, the cells at a concentration of 1 × 10⁷ CFU mL^−^¹ were resuspended in the same buffer supplemented with 1 µM DiSC_3_(5) (Sigma-Aldrich). A total of 100 µL aliquot of this suspension was pipetted into each well of a Corning 96 well black polystyrene microplate (Sigma-Aldrich). Initial fluorescence readings were taken every 5 min for 20 min in a multimode microplate reader (CLARIOstar Plus, BMG Labtech) using excitation at 622 nm and emission at 670 nm until signal stabilization. Then, 100 µL of a twofold concentrated TB_KKG6K solution was added to each well, resulting in a final concentration of 0.5×, 1×, 2×, and 4× IC_90_. HEPES buffer was used in place of the peptide solution for the untreated control. The microplate was immediately returned to the reader, and fluorescence intensities were recorded every 5 min for 20 min at 37°C. The fluorescence values obtained post-compound addition were background corrected using measurements from HEPES buffer without cells containing 1 µM of DiSC_3_(5). All samples were prepared in triplicate, and the experiments were performed at least three times.

### Membrane permeability assay

*S. aureus* cells were washed three times in PBS and diluted to a concentration of 1 × 10⁸ CFU mL^−1^. A total of 190 µL of the cell suspension, containing 7.5 µM PI (Sigma-Aldrich), was added to each well of a Corning 96-well black polystyrene microplate (Sigma-Aldrich). Baseline fluorescence was measured using excitation at 535 nm and emission at 617 nm every 5 min for 15 min in a multimode microplate reader (CLARIOstar Plus, BMG Labtech) set to 37°C until signal stabilization. Subsequently, 10 µL of TB_KKG6K was added to the respective wells to achieve final concentrations of 0.5×, 1×, 2×, and 4× IC_90_. PBS was used in place of compounds for the untreated control. Fluorescence intensities were recorded at 5 min intervals for 20 min. All samples were prepared in triplicate, and the experiments were conducted at least three times.

### *G. mellonella* toxicity assay

Groups of 20 larvae (weighing 0.3–0.4 g; SAGIP, Italy) were placed in wood shavings in a dark environment at 18°C for 24 h prior to the experiment. The larvae were then injected with 20 µL of a peptide solution (8 and 80 µM, corresponding to 1× IC_90_ and 10× IC_90_, respectively) prepared in sterile ddH_2_O. Two control groups were included: larvae injected with 20 µL of sterile ddH_2_O and untouched larvae. The survival rate was monitored for up to 6 days at 37°C.

### 3D HEEs

The 3D HEEs were purchased from Phenion (epiCS; Henkel AG, Düsseldorf, Germany). The epiCS models are 0.9 cm in diameter and constructed from human keratinocytes, isolated from juvenile foreskin obtained from a human donor. The epiCS were shipped and used for experiments on ALI 8. Upon arrival, the HEEs were immediately transferred in a standing position to a CytoOne six-well plate (Starlab, Hamburg, Germany) containing 1 mL per well of epiCS culture medium without antibiotics (Henkel AG), and equilibrated—if not otherwise stated—for 2 h at 32°C in 5% CO_2_. After specific treatments, the models were removed from the inserts and analyzed accordingly. Three HEEs (*n* = 3) were used for each experimental setup. During the experiments, the medium was changed daily. The CCM was tested for microbial contamination by plating 50 µL in duplicates on LB agar and SBA and incubating at 37°C for up to 96 h. The remaining CCM was stored at −80°C for cytokine analysis.

### Skin irritation potential of TB_KKG6K in HEEs

A skin irritation assay using MTT was conducted in accordance with the test guidelines 439 of the OECD (41). This was carried out as described previously (9) with minor changes. Following equilibration o/n, 52 µg of TB_KKG6K in 30 µL ddH_2_O was applied to the models and evenly distributed using a sterile glass applicator. The same procedure was then repeated with D-PBS (negative control) and 5% (wt/vol) SDS (Sigma-Aldrich) in ddH_2_O (positive control), respectively. All conditions were tested in triplicate. Following a 20-min incubation at room temperature (RT), each HEE insert was rinsed thoroughly 10 times with D-PBS. Subsequently, the HEEs were submerged and swirled three times in D-PBS, followed by a further 10 rinses. Following a final rinse of the exterior of the insert, any excess D-PBS was removed by gentle shaking and blotting of the insert onto sterile paper. The HEEs were then transferred into 6-well plates containing fresh epiCS culture medium and incubated for 24 h at 37°C in 5% CO_2_. The medium was then changed, and the HEEs were incubated for a further 18 h. To test the metabolic activity of the treated HEEs, they were transferred to 24-well plates (Starlab) containing 300 µL of MTT (1 mg mL^−1^; Sigma-Aldrich) in epiCS MTT Assay Medium (Henkel AG). The HEEs were incubated for 3 h at 37°C in 5% CO_2_, after which the medium was removed and 2 mL of isopropanol was pipetted into each insert, ensuring complete coverage of the tissue equivalent from both sides. Following a 2-h incubation at RT on a rotary shaker at 250 rpm, an injection needle was employed to puncture the insert membrane, allowing the tissue extract to flow into the well from which the insert was taken. Subsequently, technical duplicates of 200 µL were taken from each extract and transferred to a 96-well microtiter plate (Thermo Fisher Scientific) for OD measurement at 570 nm using a multimode plate reader (CLARIOstar Plus, BMG Labtech). Isopropanol was used as a blank. To evaluate the skin irritating potential, the relative metabolic activity was calculated. The negative control (treated with D-PBS) was assigned a metabolic activity of 100%, and the values were presented as the percentage of the mean OD_570_ (mean ± SD).

### Bacterial infection of HEEs

HEEs were infected with 3,000 CFU in PBS in a volume of 18 µL at ALI 8 using sterilized round glass applicators for even distribution. The viability of the inoculum was controlled in each infection experiment by plating the bacterial suspension used for infection in appropriate dilutions on TSA plates and counting the CFU after an incubation at 37°C for 24 h. The HEE models were incubated for 24 h (ALI 9) and 48 h (ALI 10) at 32°C in 5% CO_2_ prior to analysis. For uninfected controls, 18 µL of PBS was applied and distributed using a sterile glass applicator.

### Treatment of HEEs with TB_KKG6K

Equilibrated ALI 8 HEEs were first topically treated with 18 µL of sterile PBS and incubated for 24 h at 32°C in 5% CO_2_. A total of 31 µg of TB_KKG6K in 18 µL ddH_2_O was applied at ALI 9 to the HEEs and spread equally using a round glass applicator. For the untreated, negative control, 18 µL of sterile ddH_2_O was applied. The HEEs were incubated at 32°C in 5% CO_2_ for the subsequent 24 h until ALI 10. To analyze the penetration depth of the peptide, TB_KKG6K-FITC was applied.

For the treatment of *S. aureus*-infected HEEs, TB_KKG6K was topically applied as described above at ALI 9. For the untreated, negative control, sterile ddH_2_O was used. The models were further incubated for 24 h at 32°C in 5% CO_2_ until endpoint analyses were performed at ALI 10.

### Determination of viable bacterial numbers in infected HEEs

The bacterial load of infected, untreated and infected, TB_KKG6K-treated HEEs was determined by counting CFUs. The respective HEEs were cut out of the inserts, and the entire tissue was transferred into a 1.5 mL tube and homogenized in 500 µL PBS using a Ø 4.9 mm micro pestle (Carl Roth, Karlsruhe, Germany). An additional 500 µL PBS was added to the homogenate, which was vortexed thoroughly, and serial dilutions were plated in triplicate onto TSA. The plates were incubated at 37°C for 24 h. Uninfected HEEs were included as a negative control.

### Analysis of the permeability barrier with LY

To analyze the permeability barrier function after peptide treatment of HEEs, a method described by Holzknecht et al. (52) was applied with slight adaptations. Briefly, 50 µL of LY CH dilithium salt (1 mg mL^−1^ in PBS, Sigma-Aldrich) was topically applied onto the respective HEEs for 2 h at 32°C in 5% CO_2_, which were subsequently washed three times with PBS before preparation for cryo-section. A model exposed to 1% (wt/vol) SDS (Sigma-Aldrich) for 1 h prior to LY application was included as a control for increased skin permeability.

### Histological analysis

HEEs were excised from the insert and placed into Peel-A-Way disposable embedding molds (Sigma-Aldrich). The molds were filled with a frozen section compound (Tissue-Tek, Sakura Finetek Europe, Alphen aan den Rijn, the Netherlands) and submerged in liquid nitrogen for rapid freezing. The models were then stored at −80°C until further processing. Using a Cryostat CM1850 (Leica Biosystems, Wetzlar, Germany) set at −20°C, the samples were sectioned into 7 µm thick slices from the basal to apical layers of the HEE. The sections were mounted onto glass slides for subsequent histological staining.

### H&E staining

H&E staining was performed as described by Holzknecht et al. (52). Briefly, tissue sections were fixed in 4% (vol/vol) paraformaldehyde (PFA), washed in PBS, and hydrated in ddH_2_O. The sections were then stained with acidic Mayer’s Hematoxylin (Carl Roth) for 5 min with agitation. After 5 min of rinsing under tap water, the samples were counterstained with eosin Y solution (0.5% in H_2_O, Carl Roth) for 30 s. Following counterstaining, the sections were dehydrated through a series of ascending alcohol concentrations, cleared with ROTI-Histol (Carl Roth), and mounted using ROTI-Histokit (Carl Roth).

### Fluorescence staining

*S. aureus* infection in HEEs was visualized by immunofluorescence staining. Briefly, the sections were fixed in acetone at −20°C for 10 min and then blocked with 10% goat serum (Thermo Fisher Scientific) for 1 h. The primary anti-*S*. *aureus* antibody (ab20920, Abcam Cambridge, UK) was applied at a concentration of 5 µg mL^−1^ and incubated o/n at 4°C. The stained sections were washed three times with D-PBS. The secondary antibody, anti-rabbit IgG–Atto 488 (18772, Sigma-Aldrich), was added at a concentration of 5 µg mL^−1^ and incubated for 1 h at RT. The sections were washed three times with D-PBS and mounted in Fluoroshield with DAPI (Sigma-Aldrich). Sections treated with LY or TB_KKG6K-FITC were fixed in 4% (vol/vol) PFA and washed with PBS before they were also mounted in Fluoroshield with DAPI for microscopic analysis.

### Enzyme-linked immunosorbent assay

CCM was collected from the HEEs every 24 h (ALI 9 and ALI 10) and stored at −80°C. The CCM was analyzed using a human IL-1α ELISA Kit (Invitrogen, Thermo Fisher Scientific), and human IL-6 and IL-8 BD OptEIA ELISA kits (BD Biosciences, San Diego, CA, USA) according to the manufacturers' instructions. Absorbance readings were taken at a wavelength of 450 nm using a multimode microplate reader (CLARIOstar Plus, BMG Labtech). CCM from uninfected and untreated models served as controls, and epiCS culture medium without antibiotics was used as a blank. Cytokine concentrations in the CCM were determined based on a standard curve.

### TEM

Four milliliters of 1 × 10^8^ CFU mL^−1^ in TSB was treated with 1× IC_90_ and 4× IC_90_ of TB_KKG6K for 1 h at 37°C with shaking at 200 rpm on a rotary shaker. An untreated control was included for comparison. Following treatment, the cells were pelleted and washed three times in PBS. The washed cells were once again pelleted in a 1.5 mL tube and fixed o/n in 2.5% glutaraldehyde (vol/vol; solution in PBS) at 4°C. The pellets were post-fixed with 1% OsO_4_, dehydrated in a graded acetone series, gradually embedded in resin, 80 nm ultrathin-sectioned, stained with lead citrate, and examined using a Zeiss Libra 120 TEM (Carl Zeiss, Oberkochen, Germany). EFTEM images were captured using a 2 × 2k high-speed camera (Troendle, Germany) and analyzed with ImageSP software (Troendle, Germany).

### Scanning electron microscopy

HEEs were removed from the media at ALI 10 and immersed in 1 mL of glutaraldehyde (2.5% in PBS, vol/vol) for fixation. After fixation for 48 h at 4°C, the HEEs were subjected to an ascending alcohol series for dehydration, comprising 50%, 70%, 80%, and 99.9% ethanol. The HEEs were dried and attached to aluminum pins. The pins were sputtered with Au (Agar Sputter Coater, Agar Scientific Ltd., Stansted, GB, UK) for one minute and then analyzed by scanning electron microscopy (JSM-6010LV, JEOL GmbH, Freising, Germany).

### HEE microscopy

Microscopic evaluation of immunofluorescence stained sections was performed using a Zeiss Axioplan fluorescence microscope (Zeiss, Oberkochen, Germany) equipped with an Axiocam 503 mono microscope camera (Zeiss). The microscope was fitted with excitation/emission filters at 365/420 nm for DAPI and 428/536 nm for LY, FITC, and Atto 488. H&E-stained samples were analyzed with a Zeiss Axioplan 2 microscope (Zeiss), equipped with an Axiocam 503 color microscope camera (Zeiss).

### Image processing

All microscopy images were processed using ZEN lite microscope software (Zeiss), ImageJ software (U.S. National Institutes of Health, Bethesda, MD, USA), and Microsoft PowerPoint (Microsoft Corporation, Albuquerque, NM, USA). The LY fluorescence signal intensity was semi-quantified using ImageJ software as described (52).

### Statistical analysis

Statistical analysis was conducted using Prism 9.1.0 (216; GraphPad Software, San Diego, CA, USA). Values are given as mean ± SD per experimental setting (*n* = 3). Statistical significance (*P ≤ 0.05; **P ≤ 0.005) was determined by Student’s *t*-test if not stated otherwise
