# Erratum for Schöpf et al., “The antibacterial activity and therapeutic potential of the amphibian-derived peptide TB_KKG6K”

**DOI:** 10.1128/msphere.00469-25

**Published:** 2025-08-15

**Authors:** Cristina Schöpf, Magdalena Knapp, Jakob Scheler, Débora C. Coraça-Huber, Alessandra Romanelli, Peter Ladurner, Anna C. Seybold, Ulrike Binder, Reinhard Würzner, Florentine Marx

## ERRATUM

Volume 10, no. 6, e01016-24, 2025, https://doi.org/10.1128/msphere.01016-24. Page 5, Fig. 2D: "Mitotic" should read "Dividing."

Page 6, legend to Fig. 2D: “mitotic" should read "dividing."

Page 6, paragraph starting "The impact of TB_KKG6K on the membrane integrity," sentence 7: "mitotic status" should read "cell division status."

Page 6, paragraph starting "The impact of TB_KKG6K on the membrane integrity," sentence 8: "mitosis" should read "cell division."

